# Radiological Features of Extramedullary Hematopoiesis in a Young Male With Beta-Thalassemia: A Case Report

**DOI:** 10.7759/cureus.63945

**Published:** 2024-07-06

**Authors:** Manasa Suryadevara, Gaurav V Mishra, Neha D Shetty, Komal Mishra, Mounika Suryadevara, Anshul Sood, Chaitanya Kumar Javvaji, Riya Yadav

**Affiliations:** 1 Radiodiagnosis, Datta Meghe Institution of Higher Education and Research, Wardha, IND; 2 Radiodiagnosis, Datta Meghe Institute of Higher Education and Research, Wardha, IND; 3 Pulmonary Medicine, Ramaiah Medical College, Bengaluru, IND; 4 Pediatrics, Datta Meghe Institute of Higher Education and Research, Wardha, IND

**Keywords:** chest x ray, x-ray skull, ct -scan, extra-medullary hematopoiesis, β-thalassemia

## Abstract

The formation of the blood elements and their maturation is called hematopoiesis. In adults, this typically takes place in the bone marrow of vertebrae, ribs, and long bones. In contrast, during fetal development, the primary sites of hematopoiesis are the spleen, liver, and the yolk sac. This process of hematopoiesis, when it occurs in sites other than the bone marrow, is called the extramedullary hematopoiesis (EMH). Extramedullary hematopoiesis usually happens in patients with blood disorders like sickle cell disease and thalassemia, where there is failure of hematopoiesis in the primary sites. Here, we present a young male with beta-thalassemia who presented with shortness of breath and palpitations for one month. This manuscript discusses the imaging findings of the EMH in our patient.

## Introduction

Hematopoiesis refers to the production of blood products and their maturation [1]. In adults, hemopoiesis typically takes place in the marrow of long bones, ribs, and vertebrae. This contrasts with the fetus, where the primary sites of hemopoiesis are the yolk sac, spleen, and liver. When the primary hemopoietic sites in adults fail, such as in cases of myelofibrosis (caused by various factors like gene mutation, exposure to radiation or chemicals like toluene, etc.) and hemoglobinopathies (particularly sickle cell disease and thalassemia), extramedullary sites assume the role of formation of blood elements [1]. Thalassemia is a hereditary blood disorder passed down through an autosomal recessive pattern marked by chronic anemia [2]. Chronic hemolytic anemia frequently induces compensatory responses, with extramedullary hematopoiesis (EMH) being the most notable [3]. Extramedullary hemopoiesis refers to the production of these blood products outside the primary sites like the bone marrow, which occurs in response to blood disorders like myelofibrosis, leukemia, lymphoma, and thalassemia. 

Extramedullary hemopoiesis typically occurs in the spleen, liver, and paraspinal regions of the thorax. However, it can also involve any tissue or organ, often presenting as a mass that mimics a neoplasm. Symptoms are usually due to the mass effect. Recognizing imaging findings consistent with extramedullary hemopoiesis is crucial, as a biopsy can rule out neoplasm and significantly change the management and prognosis [1].

Here, we present a case of a young male patient with transfusion-dependent beta-thalassemia who came with shortness of breath and palpitations for 1 month.

## Case presentation

**Figure 1:**
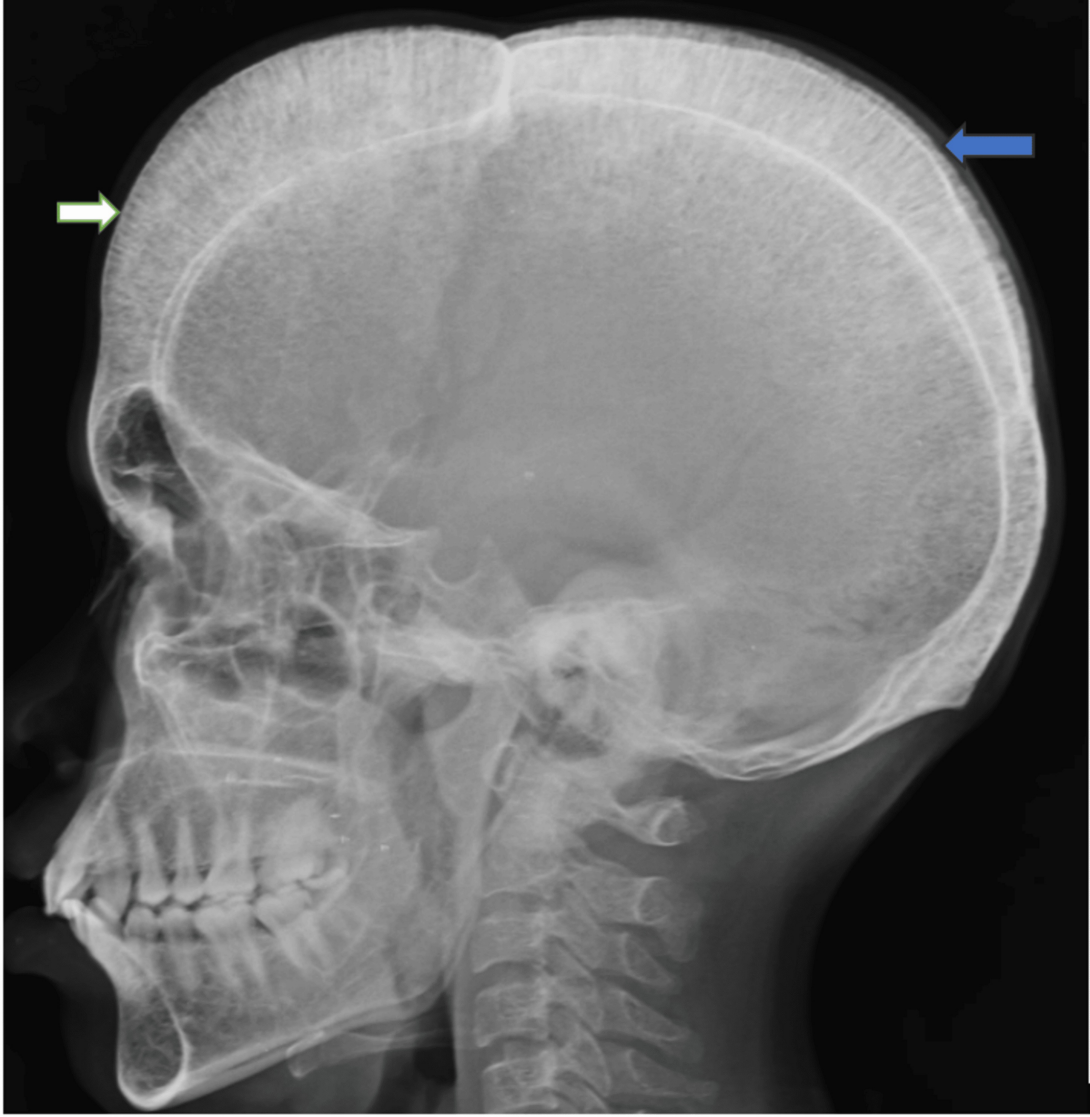
Skull X-ray lateral view showing widened diploic space of frontal (white arrow) and parietal (blue arrow) bones giving a hair-on-end appearance

**Figure 2:**
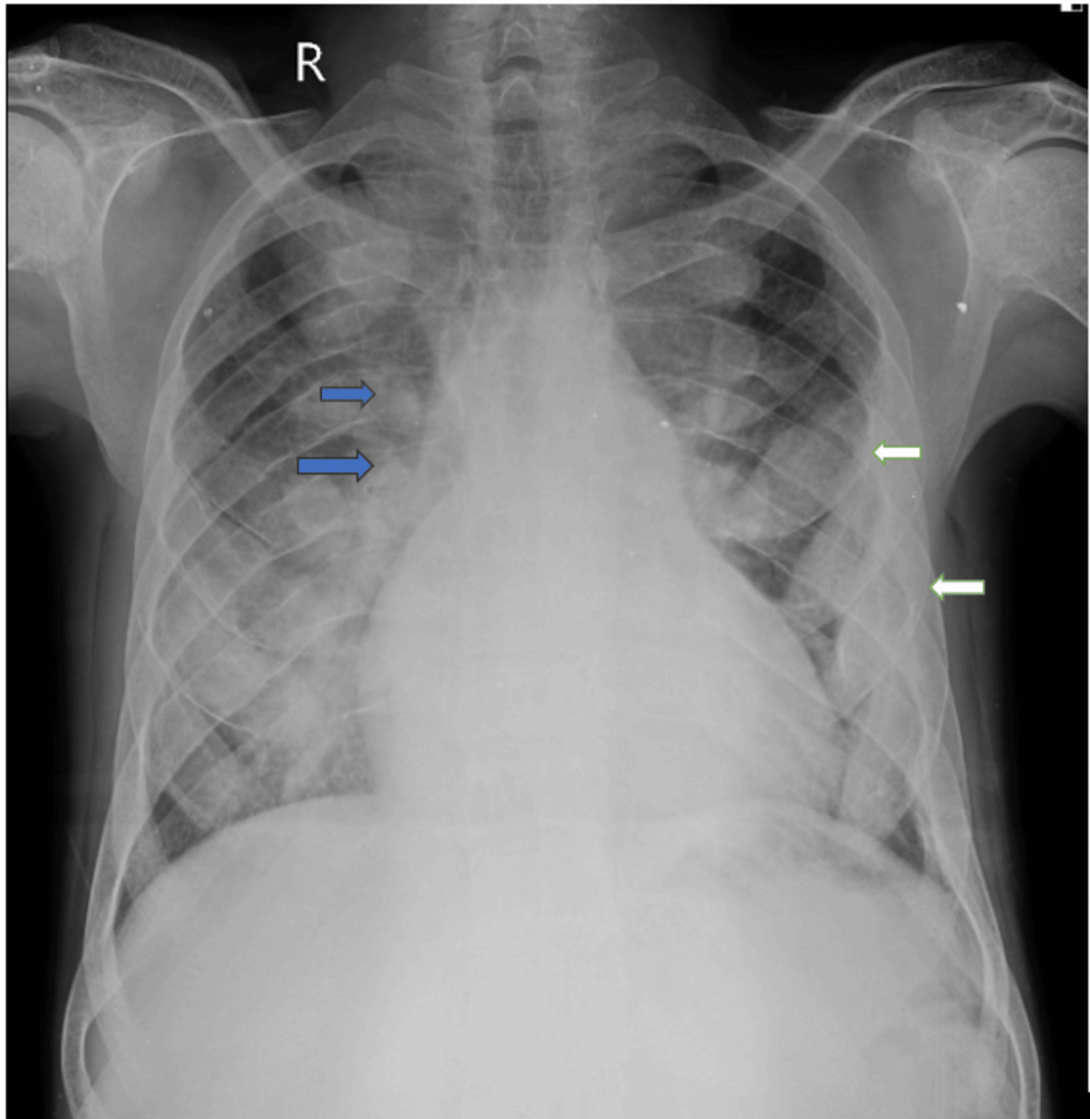
Chest X-ray posteroanterior view showing the expansion of multiple ribs at the costo vertebral junctions (blue arrows) and anterior aspect of the ribs (white arrows).

A young male patient aged 27 years, who is a known case of beta-thalassemia, is dependent on blood transfusions, and has been receiving folic acid tablets daily, was referred to our center for complaints of chest pain and palpitations. The symptoms started a month ago and were initially relieved by rest. However, the symptoms began worsening over the past 10 days. The patient had undergone splenectomy eight years ago. The patient has not been receiving blood transfusions regularly. The patient’s red blood cell count was 3.32 x 10^12^/L (4.3 - 5.8 x 10^12^/L), and hemoglobin level was 7.3 g/dl (14 - 18 g/dl) on admission. The peripheral smear showed raised eosinophils. The patient was taken up for an X-ray skull, which revealed a widened diploic space and classic hair-on-end appearance. The outer and inner tables appear thinned out (Figure 1). The chest X-ray showed expansion of multiple ribs at the costo vertebral junctions and anterior aspect of the ribs (Figure 2).

**Figure 3:**
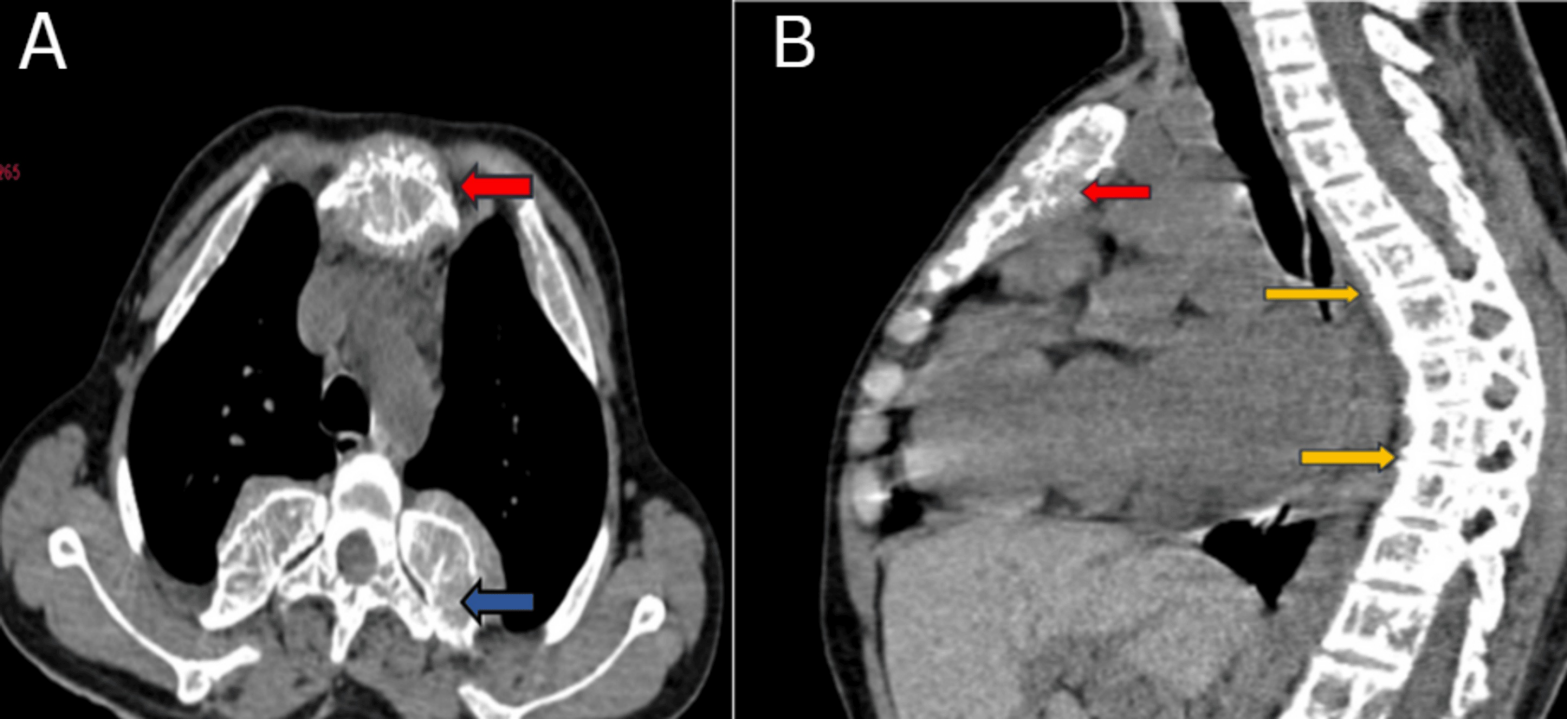
CT thorax - (A) axial section showing expanded sternum (red arrow), ribs (blue arrows) and (B) kyphotic deformity of the spine with reduced intervertebral disc spaces (yellow arrows)

**Figure 4:**
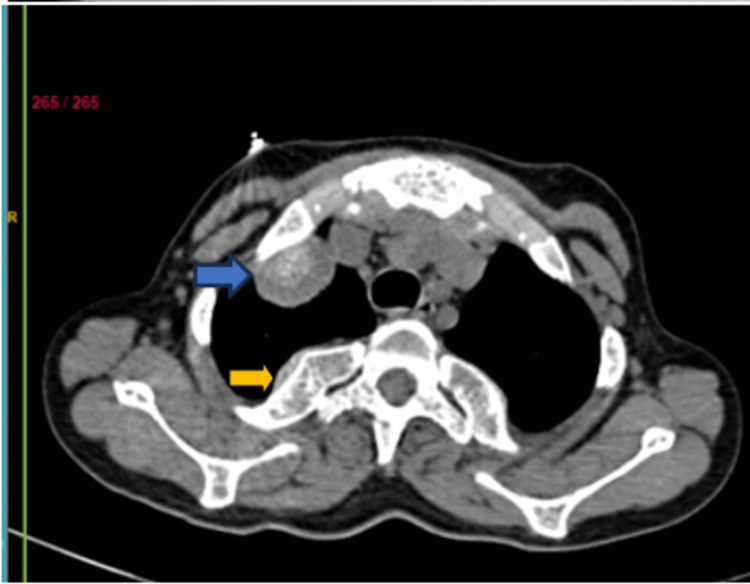
CT thorax axial section revealed diffusely expanded vertebrae (yellow arrow) and soft tissue mass (blue arrow) along the anterior end of the ribs.

**Figure 5:**
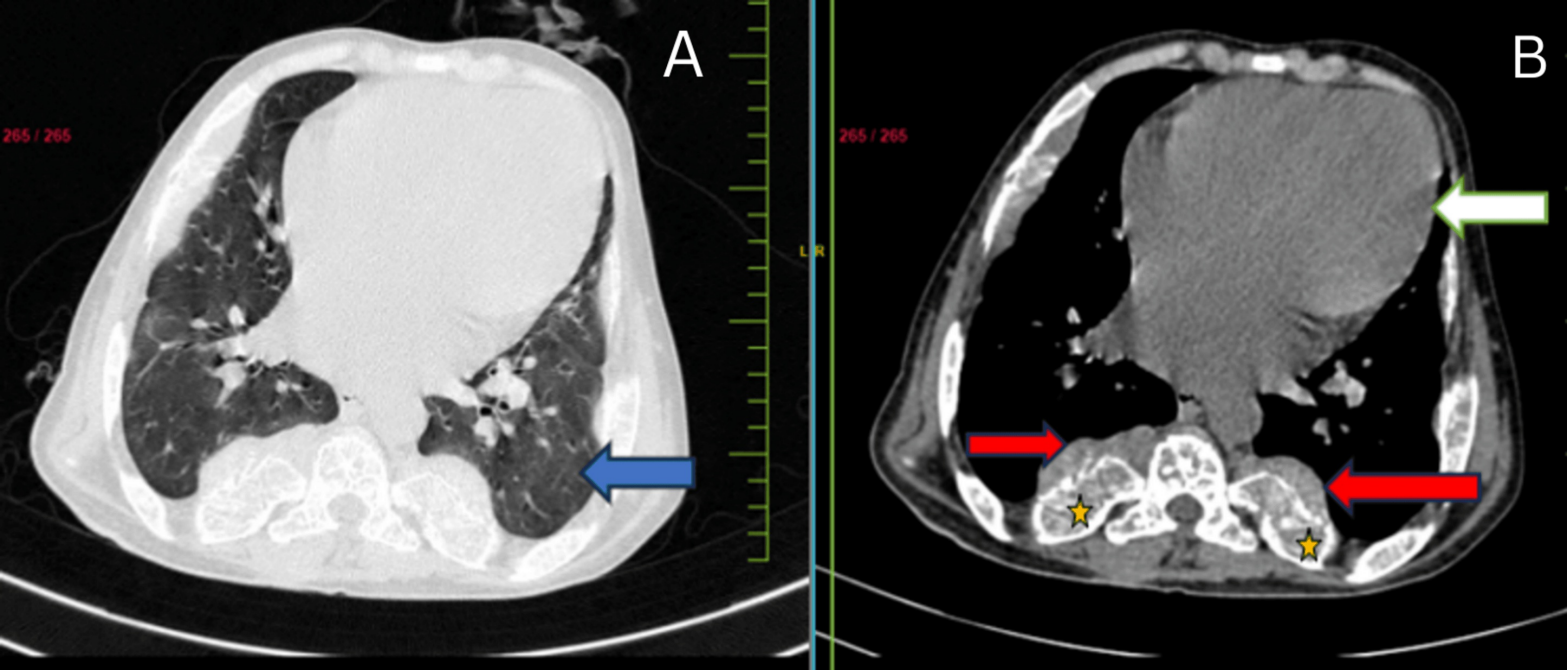
CT scan axial section - (A) Lung window: Lungs show diffusely decreased lung volume (blue arrow); (B) Mediastinal window showing enlarged heart suggesting cardiomegaly (white arrow) and expanded ribs (star) with paraspinal soft-tissue masses adjacent to them

A CT scan revealed diffusely expanded vertebrae, ribs, and sternum (Figure 3), with multiple well-defined para-osseous smooth lobulated soft tissue density masses noted along bilateral paravertebral/posterior mediastinal, subpleural regions, along the anterior ends of ribs suggestive of (s/o) thoracic extramedullary hematopoiesis. The largest soft tissue density mass was noted adherent to the first right rib measuring 3.2 x 1.8 cm in subpleural space anteriorly (Figure 4). Lungs showed decreased volume diffusely. Cardiomegaly was noted (Figure 5).

**Figure 6:**
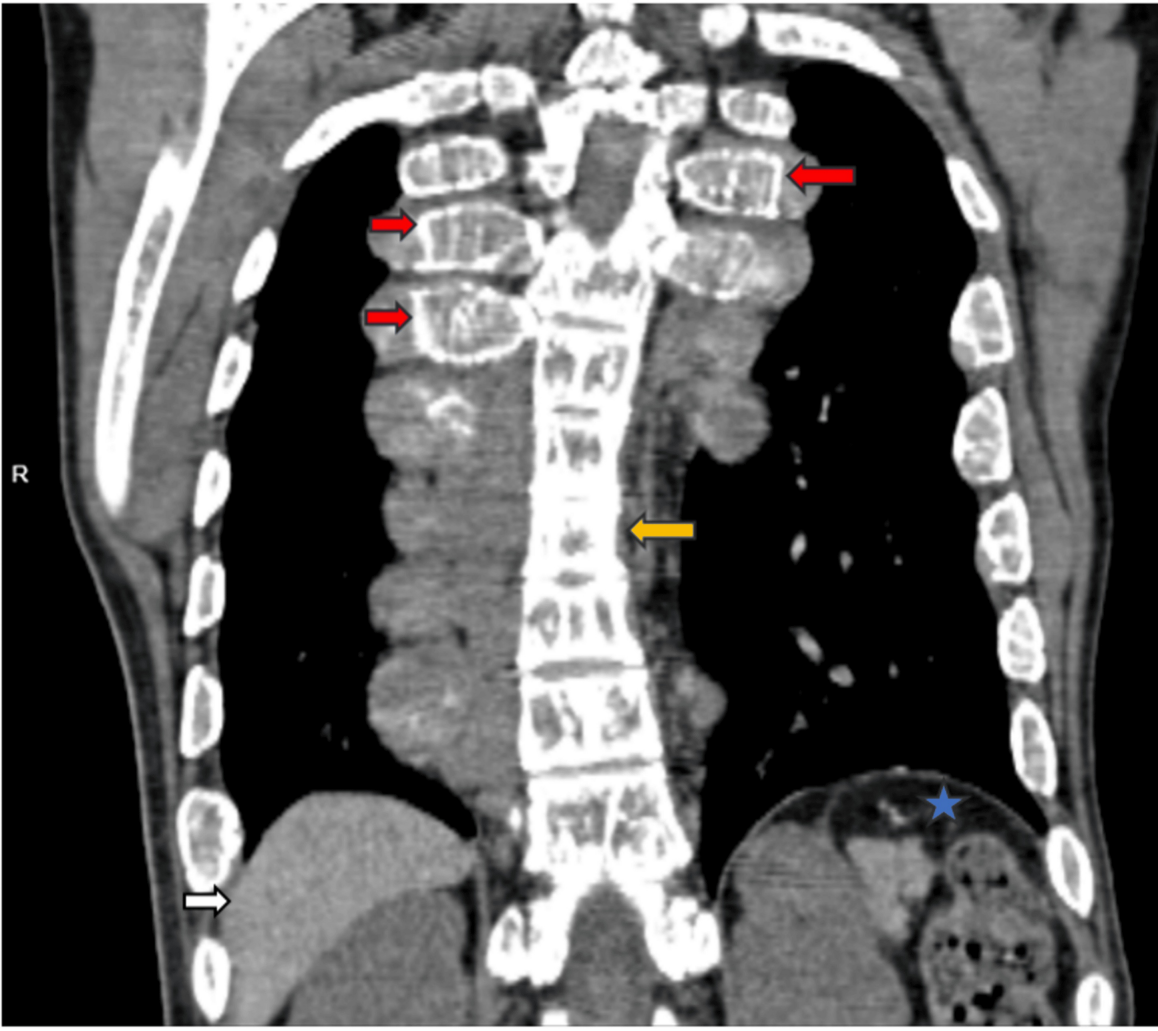
CT coronal section showing kyphotic deformity of the dorsal spine with loss of bony trabeculae (yellow arrow) and reduced intervertebral disc spaces. Bilateral ribs appear expanded (red arrows). The visualized part of the liver shows diffuse hyperattenuation (white arrow). Spleen was not visualized in the left hypochondrium, which is consistent with post spleenectomy (star).

Kyphotic deformity of the dorsal spine with loss of bony trabeculae and reduced intervertebral disc spaces were noted. The visualized part of the liver shows diffuse hyperattenuation, suggesting secondary hemochromatosis. The spleen was not visualized in the left hypochondrium, which is consistent with post-splenectomy status (Figure 6). 

**Figure 7:**
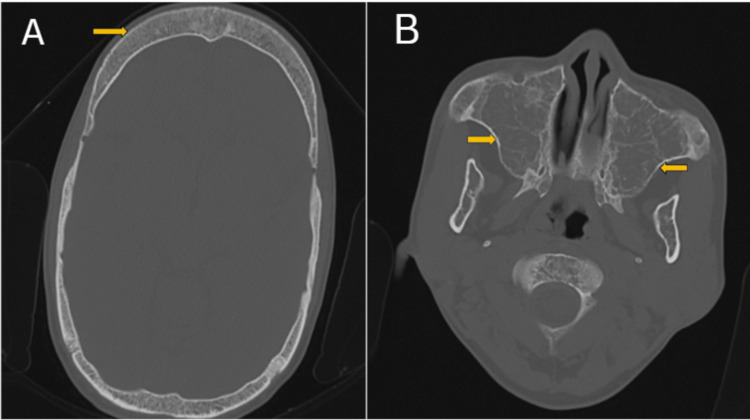
CT Temporal bone axial section showing (A) widened diploic space of frontal bone (arrow) and (B) Hyperplasia and consequent dilatation of bilateral maxillary sinuses with decreased wall thickness (arrows).

The patient has undergone one blood transfusion because of low hemoglobin, following which the hemoglobin level rose to 8.3 mg/dl. The patient underwent high-resolution computed tomography (HRCT) temporal bone as advised in view of pain in the right ear. The scan suggested otitis media of the right ear and showed a widened diploic space (Figure 7A). The bilateral maxillary sinuses showed hyperplasia and consequent dilatation with decreased wall thickness (Figure 7B).

Following this, the patient underwent a bone marrow biopsy, which revealed eosinophilia with hemolytic features, erythroid hyperplasia, with early megaloblastic change. The findings were compatible with the diagnosis of extramedullary hematopoiesis. The patient has been advised of blood transfusions, steroids, and radiation therapy. The patient refused further stay and hence was discharged against medical advice with a prescription of medications.

## Discussion

Hematopoiesis is the production and maturation of elements of blood, which typically takes place in the vertebrae, ribs, and marrow of long bones in adults, while in the fetus, the principal sites of hemopoiesis are the liver, spleen, and yolk sac [1]. Few hematopoietic stem cells (HSCs) circulate in the spleen and peripheral blood normally [4]. When these primary sites in the adult fail like in a few blood disorders and the patients do not receive indicated blood transfusions, to overcome the resultant anemia, the bone marrow gets ramped up leading to osteoporosis and deformities in bone [1][4]. Consequently, erythroid precursors migrate from the bone marrow to other organs. This process of hematopoiesis outside the primary sites is called the extramedullary hematopoiesis (EMH) which commonly occurs in the spleen, liver, and paraspinal regions of the thorax. However, beyond these typical sites, it can involve virtually any tissue or organ, often presenting as a mass that mimics a neoplasm [1].

Paraspinal extramedullary hematopoiesis (EMH) is incidentally found and asymptomatic in more than 80% of cases. However, when it does present with symptoms, it can be debilitating due to its compressive effects on neural tissue [5].

Extramedullary hematopoiesis (EMH) often manifests typically as paraspinal masses in the chest, found incidentally. These masses are commonly associated with thalassemia and appear as bilateral soft-tissue masses with a smooth surface, having areas showing fat attenuation on computed tomography (CT) [6].

EMH on CT typically shows hypovascular, heterogeneous soft tissue mass lesions interspersed with fat density areas [1]. On sonography, EMH can present as solid masses with internal vascularity. Magnetic resonance imaging (MRI) typically reveals heterogeneous masses with variable T1 and T2-weighted appearances, often containing lipid components with variable enhancement [6]. Paraspinal EMH can occur at sites other than primary hematopoietic sites, possibly due to marrow extrusion through thin vertebral body cortices, a common finding in patients with hemoglobinopathies. Their prevalence is higher in the thorax compared to the abdomen or pelvis [6]. Rib expansion is another common thoracic manifestation, particularly in thalassemia patients [1].

The majority of the patients (63%) with non-hepatosplenic extramedullary hematopoiesis present with symptoms specific to the site involved. The rest are either asymptomatic or present with generalized symptoms such as fatigue. Apart from hepatosplenomegaly, the most common manifestation of extramedullary hematopoiesis is bilateral, fat-containing, heterogeneous paravertebral masses. In these cases, a biopsy is typically not necessary [6].

Extramedullary hematopoiesis rarely occurs in transfusion-dependent thalassemia major, with an incidence rate of only 1% [7]. This discrepancy in prevalence between non-transfusion-dependent and transfusion-dependent thalassemia is likely due to transfusions suppressing the need for EMH to compensate for anemia in the latter [3].

In patients with beta-thalassemia, common imaging abnormalities include widening and a distinctive trabeculated pattern caused by osteoporosis affecting the ribs in their entire length [8]. The ribs appear as though another rib is superimposed, giving a "rib within the rib" appearance, a phenomenon typically seen in the anterior and middle parts. This occurs due to inadequate hematopoiesis, where the subperiosteal red bone marrow extends longitudinally within the cortex, leading to a compensatory expansion of the bone marrow [9]. Additionally, well-defined, small (1-2 mm) localized lucencies in the medulla may be present. It is believed that initiating a hypertransfusion regimen early in life can prevent changes in the ribs [8].

Patients with intermediate beta-thalassemia typically exhibit symptoms that fall between those of carriers and individuals with major beta-thalassemia, which include moderate anemia, which may necessitate intermittent blood transfusions, albeit at longer intervals compared to patients with major beta-thalassemia. Complications arising from hemolysis and ineffective erythropoiesis, such as pain, pulmonary hypertension, and peptic ulcers, are also common [8].

This manuscript presents a case of intermediate beta-thalassemia with the widening of diploic space, ribs, and paraspinal masses in the thorax, which are infrequent sites for extramedullary hematopoiesis (EMH).

Radiographic imaging is the primary diagnostic tool of utmost significance though the history and examination can provide valuable perception, which broadens the differential diagnosis and confirms the presence of hematopoietic tissue. On MRI, the lesions may be apparent, often revealing significant deposition of iron in individuals receiving blood transfusions. The inactive, older lesions are marked by the deposition of iron and fat whereas active hematopoietic lesions usually show vigorous neovascularization [10].

To suppress the production of erythropoietin, treatment options for this condition comprise multiple blood transfusions and radiotherapy to hinder the overgrowth of marrow tissue. Surgical decompression is another approach advised. The advantages of these treatments remain relatively unclear in this condition due to their rarity. For reducing inefficient hematopoiesis, adequate blood transfusions are crucial in thalassemic patients, thus preventing the extensive development of EMH. It is vital to anticipate and prevent complications associated with hypertransfusion therapy, which are common. In hypertransfusion therapy, the target hemoglobin (Hb) level is usually set above 10 g/dL [10].

## Conclusions

Extramedullary hematopoiesis (EMH), though rare, can occur in patients with transfusion-dependent thalassemia (TDT) and lead to significant clinical complications. In TDT patients, EMH tends to present at a higher age compared to those with non-transfusion-dependent thalassemia, with the spine being the most common site, though various organs can be affected. Management of EMH in TDT involves both invasive and conservative approaches, yet there is no comprehensive comparison of their outcomes. Given the increasing incidence of EMH in TDT patients, it is crucial to study this phenomenon broadly, focusing on management strategies. Physicians should aim to maintain pre-transfusion hemoglobin (Hb) levels of at least 9 g/dL to prevent EMH and actively monitor for it in TDT patients, particularly those unable to achieve the recommended Hb levels, ensuring early detection and individualized treatment plans for effective management.
